# What You Believe Can Affect How You Feel: Anger Among Caregivers of Elderly People With Dementia

**DOI:** 10.3389/fpsyt.2021.633730

**Published:** 2021-04-07

**Authors:** Haoran Wang, Hongmei Cui, Meng Wang, Chunyan Yang

**Affiliations:** ^1^Dalian Seventh People's Hospital, Dalian, China; ^2^Qindao Mental Health Center, Qingdao University, Qingdao, China

**Keywords:** elder, dementia–Alzheimer disease, caregiver, anger, epidemiological

## Abstract

**Background and Purpose:** Anger has been recognized as a commonly experienced emotion among caregivers of elderly people with dementia. While several cognitive behavioral therapy (CBT)-based intervening methods have been developed, limited research has systematically examined the associations between dementia-related cognition and caregiving anger. Currently, we focused on three representative and well-studied cognitive constructs, *person-centered attitude* (PCA), *dementia representation* (DR), and *empathy*, exploring how they related to caregiving anger.

**Methods & Results:** In total, 327 caregivers (239 female) participated in the study and finished online questionnaires. Multi-variable regression analyzes showed that PCA (β_PCA_ = −0.22^**^) and empathy (β_empathy_ = −0.18^**^) could negatively predict caregiving anger. However, all DR dimensions had no influence on caregiving anger except *coherence* (β_coherence_ = −0.24^**^) in the current study.

**Conclusion:** Generally, lower caregiving anger was associated with: (1) being more empathic; (2) having a person-centered attitude; and (3) having a comprehensive understanding of dementia. The results of this study provide detailed suggestions for the development of anger management programs for caregivers of people with dementia.

Dementia, referring to a clinical syndrome characterized by progressive deterioration in cognitive ability and the capacity for independent living (1), affects more than 4% of people aged 60 years or over worldwide (5.21% in China). And due to aging of the population worldwide, the number of patients with dementia is estimated to triple in the next two decades (2). The rapid increase in prevalence of dementia will generate a substantial burden on health care systems and the local economy. Data showed that providing dementia patients with health care costs nearly US $ 30 billion in the UK per year, and this number is projected to rise in the future (3). In China, experts estimated that annual health care costs directly associated with dementia are supposed to reach $ 1 trillion (4). Considering the rising demand for formal health care and relatively limited public health resources, informal care needs to serve as an important supplement. It is reasonable to foresee that more and more patients with dementia will live within the community and receive unpaid care from their relatives (i.e., informal caregivers).

However, many caregivers do not choose their role. Previous research has shown that caregivers could find it difficult to adapt to caregiving due to emotional distress and burnout (5). In fact, caring for patients with chronic disease has long been identified as a resource of stress which could bring about various negative consequences (6). Anxiety and depression are two of such consequences which have received much research attention (7). However, several studies have shown that anger is also commonly experienced among family caregivers and this emotion response has largely been overlooked (8, 9). This lack of interest could be attributed to caregivers' difficulty in acknowledging their anger (9).

Fortunately, several intervention methods have been designed to help caregivers manage the anger they experience during caregiving. For example, psychoeducation programs in the form of a face-to-face course or video have been developed (10, 11). These programs can be categorized as following: (1) skill training. These courses aim to teach participants cognitive skills in dealing with their feelings, such as relaxing techniques (i.e., distraction techniques) and awareness training (11); (2) cognitive behavioral therapy (CBT)-based methods. These courses provide trainees with cognitive change strategies such as cognition reconstruction (12) and stopping thoughts which commonly precede anger (13).

However, previous meta-analyses indicated that these intervention programs could be less efficacious than researchers have expected. Gallagher and his colleagues (14) reported an averaging effect size of 0.15 based on 19 interventions studies. This indicated relatively low effectiveness for existing intervening methods to alleviate caregivers' psychological dysphoria. The low effectiveness could partly be attributed to the lack of insights on the cognitive bases which may lead to anger (14). According to Beck's cognitive behavior model (15), one's cognitive explanation about an event can influence his or her behavioral and emotional reactions to it. Thus, caregivers' disease-related cognition may determine the feelings (including anger) they experience during caregiving. There has been some initial evidence which could support this assumption. For example, Lo Sterzo's (16) research found that caregivers' comprehensibility of dementia could influence the level of depression they experienced during caregiving. Likewise, Shinan found that caregivers with higher levels of dementia-related knowledge were less likely to experience burnout conditions (17).

Although all the intervention paradigms mentioned above are expected to target caregivers' cognitive systems, the relation between caregivers' cognition about dementia and anger experiences have not been clearly explored. According to the common sense model [CSM, (18)], beliefs, attitudes, and cognitive ability are the three essential constituents of disease-related cognition. Holding together several theoretical threads, we assumed that three representative, well-studied cognitive conceptualizations could be related to anger during caregiving, including (1) dementia representation (DR, beliefs about dementia); (2) person-centered attitudes (PCA, a widely recommended attitude toward patients with dementia); and (3) empathy (an essential cognitive ability when dealing with people with dementia).

First, dementia representation (DR), which reflects how caregivers understand dementia, could be possibly related to caregiving anger. Quinn's (19) research found that the caregivers who described their patients as “being forgetful” and attributed the symptoms to aging were less likely to go through depression than those who regarded the care-receivers as “having an incurable disease.” According to REDIX (19), the way caregivers name the disease is referred to as “identity,” which is one dimension of DR. Likewise, Lo Sterzo and Colleagues (16) found that coherence, one dimension of DR, was associated with caregivers' general well-being. Thus, it is reasonable to assume that how caregivers perceive dementia-related problems may affect caregiving anger.

Second, person-centered attitudes (PCA) (20), characterized by regarding patients as individuals with *personhood and dignity*, could also be related to anger in caregivers. Previous findings indicated that after the caregivers received training on person-centered care, their care-receivers showed lower levels of psycho-dysphoria (21). Evidence also showed that being cognitively impaired does not necessarily reduces one's perception of the way in which he/she is treated (21). It is reasonable to assume that viewing patients as people with an intact personhood and self-esteem may reduce the conflicts between caregivers and patients, and subsequently lead to lower levels of anger for caregivers.

Finally, empathy is defined as “an ability to place oneself mentally and emotionally in the world of another person, to apprehend another's condition and state of mind, to communicate understanding back to the other and perceive his reaction to it” (22). Previous evidence showed that empathy may also play an essential role in improving caregiving outcomes and alleviating negative feelings (such as depression and anxiety) during caregiving (23). It is reasonable to assume that being empathic may alleviate caregiving anger.

In summary, the aim of the current research is to explore systematically how caregivers' disease-related cognition (DR, empathy, and PCA) may influence their anger experiences during caregiving.

## Methods

### Participants

The participants consisted of 327 dementia caregivers, who were recruited from two local dementia/neurological centers using Weixin, a smartphone application used for online communication. One important function of Weixin is “group chat” by which people (<500) can chat with each other simultaneously. In the Chinese medical system, a large amount of physicians utilize such “group chats” to manage and send out information simultaneously to all of their patients (equivalent to online bulletin boards). A total of 10 psychiatrists/physicians participated in the current study, and the recruitment advertisements (including the main conductor's phone number) were sent out in each psychiatrist/physician's Weixin group chat. A total of 340 caregivers joined the study and 327 of them finished the online questionnaire. The main inclusion criteria were: (1) caregivers were identified as family members/friends of patients and the care he/she provided was not paid; (2) patients were diagnosed with dementia according to the International Classification of Disease, Tenth Edition (ICD-10) and lived within the community (not living in or receiving care from professional organizations). And the main exclusion criterion was: caregivers were paid. By this criterion, formal caregivers (care workers) were excluded.

### Procedure

The research was approved by the Ethnic Committee of Dalian's Seventh People Hospital (DLS20200812). All the assessments were conducted by trained graduate students. First, participants were read an institutional review board (IRB)-consented script and gave vocal consent before participating in the study. All responses from the participants were recorded carefully and stored safely in an online file.

### Measurements

DR was measured by the brief illness presentation questionnaire (Brief IPQ) developed by Lo Sterzo (16). Considering the questionnaire had not been localized using the Chinese population, a double translation method was adopted to make sure the items were suitable for a Chinese sample. Results showed that all items except IPQ-5, “how much do you experience symptoms from your relative's illness” could be double translated appropriately. We further interviewed 10 participants to examine whether the Chinese version of the Brief IPQ made sense to Chinese caregivers. And the results showed that half of them (5) found IPQ-5 weird, for the reason that “usually they don't think in that way.” Regarding this, IPQ-5 was deleted in the version we finally adopted. As all items in the Brief IPQ are independent, the deletion of one item cannot affect the others.

PCA was measured by one subscale of the approach to dementia questionnaire [ADQ, (24)], which is widely used to evaluate to what extent caregivers agree with the idea that “people with dementia are unique individuals with the same values as others.” Both a previous study and the current study showed good reliability (*α* = 0.83; *α* = 0.89 for the current research).

Empathy was measured by the “perspective-taking” subscale of the interpersonal reactivity index (PT-IRI) (25), which is a widely used instrument to evaluate adults' tendency to adopt the psychological perspective of someone else. In the current study, “others” in the items were re-phrased as “dementia patient” but the response keys were kept unchanged. In the current study, PT-IRI showed good reliability (*α* = 0.94).

Anger related to caregiving was measured with a caregiver anger interview (11), which was developed specifically for measuring caregivers' anger intensity. Participants were asked to write down three most annoying events in the last 2 weeks which were directly or indirectly related to caregiving. And then they were asked to rate the three events across six anger adjectives (aggravated, angry, annoyed, disgusted, frustrated, and resentful). The measurement showed good reliability in the current study (*α* = 0.91).

### Data Analysis

Descriptive statistics and multi-variable regression analyses were conducted using SPSS 19.0. Missing values (<5%) were substituted with the average value for the subscale. As more than 95% of caregivers perceived dementia as “lasting forever,” the item about *timeline* was deleted from further analyzes.

## Results

### Participants' Characteristics

The majority of the 327 caregivers were female (73.1%), married (81.7%), and middle-school-level educated (71.4%). More than half of the care receivers were diagnosed as having “Alzheimer's disease” (55.7%), and most of them had a long disease course (>3 years). Participants' demographic information is listed in Table 1.

**Table 1:** Participants' characteristics.

**Demographic information**	***N* (%)**
Characteristics of caregivers	
Gender	
Female	239 (73.1%)
Marriage	
Married	267 (81.7%)
Unmarried	45 (13.8%)
Divorced	15 (4.6%)
Age	
<65	238 (72.8%)
Kin-relationship	
Spouse/partner	98 (30%)
Adult children	194 (59.3%)
Other	35 (10.7%)
Education	
Middle-school level	242 (71.4%)
College-level and above	85 (28.6%)
Characteristics of care-recipients	
Diagnosis	
Alzheimer's disease	182 (55.7%)
Mixed Alzheimer's and vascular dementia	51 (15.6%)
Vascular dementia	32 (9.8%)
Unspecified/other dementia	31 (19%)
Time since diagnosis	
<1 year	100 (30.6%)
1–2 years	60 (18.3%)
3–5 years	167 (51.1%)

### Sociodemographic Variables and Caregiving Anger

Subgroup analysis was conducted to evaluate the effects of demographic variables on caregiving anger. *T*-test showed that female caregivers had higher levels of anger (*t* = −3.71, *p* < 0.001). Results from one-way ANOVA found that characteristics of both caregivers (such as education level) and patients (dementia subtypes) had no effect on caregiving anger.

### DR and Caregiving Anger

The mean score and deviation of every single dimension of DR are listed in Table 2. All seven dimensions of DRs were related to caregiving anger (*r*_consequences_ = −0.55; *r*_personal control_ = −0.62; *r*_treatment control_ = −0.53; *r*_concern_ = −0.57; *r*_coherence_ = −0.65; *r*_emotional representation_ = −0.33). Then, a multiple regression analysis was conducted to evaluate how well the DRs predicted caregiving anger, adjusting for the covariate (gender). The results revealed that only *coherence* had a predictive effect on caregiving anger (β= −0.24^**^; *t*-value = 4.29).

**Table 2:** Mean scores and deviations of every dimensions of DR.

**Dementia representation**	**Means**	**S.D**.
Consequences	6.00	1.22
Personal control	3.67	0.76
Treatment control	2.64	2.44
Concern	3.87	1.17
Coherence	4.65	1.13
Emotion representation	3.71	1.05

### Empathy, PCA, and Caregiving Anger

The result showed that empathy and PCA were also significantly associated with caregiving anger (*r*_empathy_ = −0.57; *r*_person−centered attitude_ = −0.60). A multiple regression analysis was also conducted to how well-empathy and PCA predicted caregiving anger. After adjusting for covariates (age, gender, and time since diagnosis), empathy and person-centered attitude still had a predictive effect on caregiving anger (β_empathy_ = −0.22^**^, *t*-value = 2.09; β_PCA_ = −0.18^**^, *t*-value = 6.09). The results from multivariate regression analysis are shown in Table 3.

**Table 3:** Dementia representation, empathy, and person-centered attitude predicting caregiving anger.

**Anger**	**β**	***t*-value**
**Dementia representation**		
Consequences	−0.07	−1.27
Personal control	−0.08	−1.52
Treatment control	−0.08	−1.11
Concern	−0.11	−1.92
Coherence	−0.24^**^	−3.96
Emotion representation	0.03	0.40
Empathy	−0.18^**^	−3.98
Person-centered attitude	−0.22^**^	−4.37

^*^P < 0.05,

** *p < 0.01*,

^***^p < 0.001.

## Discussion

This is the first study to investigate anger among Chinese caregivers of patients with dementia. A total of 66% of caregivers reported having experienced moderate to high levels of anger in caregiving situations. Considering previous research findings (9, 26), more than half of family caregivers reported anger-related problems. This stresses the necessity of developing effective anger management methods for caregivers of people with dementia. Consistent with previous findings (27), female caregivers showed higher anger intensity, which is probably due to their propensity to be “emotionally involved” (28). Highly involved caregivers are more likely to have their emotions affected by patients and thus can more easily burnout. Compared to female caregivers, male caregivers are disassociated from the patients whom they take care of Jutten et al. (23).

The current research is also the first to systematically explore the effects of caregivers' dementia-related cognition (beliefs, attitude, and cognitive ability) on anger they experience during caregiving. Being more empathic did lead to lower caregiver anger intensity as expected. Actually, abundant evidence has shown that cognitive empathy (i.e., perspective taking) is reversely related with anger. Research by Oliver and colleagues (29) indicated that individuals with higher levels of empathy were more aware of others' distress and were more concerned with negative consequences caused by them if they expressed their anger. That is, high-empathy individuals are better at anger control. Besides, Jutten and her colleagues (30) found that empathy may help caregivers recognize dementia symptoms as part of disease presentations rather than intentional acts of the patients. This may possibly reduce caregivers' anger during caregiving. However, research showed that being empathic consumes more cognitive resources (31) and may add to psychological burden (23). For high-empathy caregivers, it is still unclear whether their low anger expression is at the expense of their general psychological well-being. The relation between caregivers' anger, empathy, and psychological well-being need further exploration.

PCA was found to be related to lower levels of caregivers' anger in the current study. In contrast to the traditional belief that people with dementia are mentally disabled, a person-centered attitude implies that caregivers regard patients as people with personhood, strengths, and normal needs (32). In other words, PCA requires caregivers to view patients as a “*whole person with cognitive impairment*.” Research showed that behavioral and psychological symptoms of dementia (BPSD), a main source of caregivers' anger and resentment, are not only the consequence of disease itself, but responses to adverse psycho-social environments (20). Caregivers with PCA are less likely to infantilize, disempower, and objectify their care-recipients, and in consequence the care-recipients showed less agitation and aggression (20). It is reasonable to assume that caregivers with PCA experience lower levels of anger for their care-recipients, and their patients show less BPSD. In addition, according to Kitwood (32), caregivers with person-centered attitudes have higher levels of self-compassion, and thus the emotional strains (such as anger) of being a caregiver are more likely to be recognized, respected, and reasonably expressed.

The current study was also the first to explore the effects of caregivers' dementia-related beliefs (DR) on their angry feelings during caregiving. The results showed that only one of the six dimensions of DR, *coherence* (i.e., “*understanding the disease well*”) was associated with lower caregiving anger. A sense of coherence is one of the core conceptions in health psychology (33), which refers to “a global and pervasive feeling that the life events one faces are comprehensible, meaningful, and worthy of engagement.” For dementia caregivers, being coherent means that he/she feels the symptoms of dementia are comprehensible and manageable to some extent. Lo Sterzo and colleagues (16) found that caregivers with a sense of coherence about dementia were prone to lower levels of caregiving-related anxiety and depression. Likewise, Quinn and her colleagues (34) found that even patients who have a general comprehension of the symptoms they experience (i.e., high sense of coherence) can deal with the impact of dementia more effectively. These findings showed that viewing symptoms as comprehensible and manageable render caregivers or patients more self-efficient in dealing with disease consequences (16), including frustration and anger. It should also be noted that items of specific knowledge about dementia (such as cause, timeline, and cure effectiveness) were not associated with lower caregiving anger in the current research. These results may reflect that being coherent requires a “feeling of understanding” rather than specific knowledge about dementia (such as knowledge about causes, disease course, etc.).

In all, in the current study we found that lower caregiving anger was associated with: (1) being more empathic; (2) having a person-centered attitude; and (3) understanding symptoms of dementia more comprehensively (i.e., being more coherent). These findings may inform the designs of anger management lectures that provide participants with the whole picture of dementia and facilitate intuitive understanding of the illness. On the contrary, providing specific disease-related knowledge and explanations may not be helpful like expected. Programs based on virtual reality (VR) technologies may be an ideal choice to help caregivers understand the perspective of people with dementia (35). It may provide a sense of what it is like to live with dementia, and such “subjective knowledge” is essential for caregivers to develop empathy and a general comprehension of dementia (35).

There are also several limitations in the current study which should be noted. First, the convenient sampling methods adopted may undermine the representativeness of the current research. The participants from two major cities of China (Qingdao, Dalian) may not represent caregivers living in other parts of China, and it should be noted when generalizing the findings. Second, a cross-sectional design did not allow the determination of causal relationships, and thus the current study may act as an initial exploration of associations between dementia-related cognition and caregiving anger. Research using longitudinal designs are needed in the future to verify these associations. In conclusion, the current study examined the associations between caregivers' dementia-related cognition (DR, PCA, and empathy) and caregiving anger. Future research is needed to examine the associations between a wider range of dementia-related cognition and caregiving anger, hoping to provide more specific and detailed guidance for designs of anger management programs.

## Data Availability Statement

The raw data supporting the conclusions of this article will be made available by the authors, without undue reservation.

## Ethics Statement

The studies involving human participants were reviewed and approved by Ethics committee of Dalian NO.7 People's hospital. The patients/participants provided their written informed consent to participate in this study.

## Author Contributions

CY proposed the research idea. HW collected the data and wrote the paper. HC and MW helped in data collection, revised the manuscript, and provided many constructive suggestions. All authors contributed to the article and approved the submitted version.

## Conflict of Interest

The authors declare that the research was conducted in the absence of any commercial or financial relationships that could be construed as a potential conflict of interest.
